# Lower comorbidity scores and severity levels in Veterans Health Administration hospitals: a cross-sectional study

**DOI:** 10.1186/s12913-024-11063-3

**Published:** 2024-05-08

**Authors:** Matthew P. Dizon, Adam Chow, Michael K. Ong, Ciaran S. Phibbs, Megan E. Vanneman, Yue Zhang, Jean Yoon

**Affiliations:** 1 Center for Innovation to Implementation, Veterans Affairs Palo Alto Health Care System, 795 Willow Road (152 MPD), Menlo Park, CA USA; 2 Department of Health Policy, Stanford University School of Medicine, Stanford, CA USA; 3 Health Economics Resource Center, Veterans Affairs Palo Alto Health Care System, Menlo Park, CA USA; 4 Center for the Study of Healthcare Innovation, Implementation, and Policy, VA Greater Los Angeles Healthcare System, Los Angeles, CA USA; 5 David Geffen School of Medicine and Fielding School of Public Health, UCLA, Los Angeles, CA USA; 6 Department of Pediatrics, Stanford University School of Medicine, Stanford, CA USA; 7 Informatics, Decision-Enhancement and Analytic Sciences Center, VA Salt Lake City Health Care System, Salt Lake City, UT USA; 8 Division of Epidemiology, Department of Internal Medicine, University of Utah School of Medicine, Salt Lake City, UT USA; 9 Division of Health System Innovation and Research, Department of Population Health Sciences, University of Utah School of Medicine, Salt Lake City, UT USA; 10 Division of Biostatistics, Department of Population Health Sciences, University of Utah School of Medicine, Salt Lake City, UT USA; 11 Department of General Internal Medicine, School of Medicine, University of California at San Francisco, San Francisco, CA USA

**Keywords:** Outcomes research, Observational study designs, Health care quality, Administrative data, Documentation

## Abstract

**Background:**

Previous studies found that documentation of comorbidities differed when Veterans received care within versus outside Veterans Health Administration (VHA). Changes to medical center funding, increased attention to performance reporting, and expansion of Clinical Documentation Improvement programs, however, may have caused coding in VHA to change.

**Methods:**

Using repeated cross-sectional data, we compared Elixhauser-van Walraven scores and Medicare Severity Diagnosis Related Group (DRG) severity levels for Veterans’ admissions across settings and payers over time, utilizing a linkage of VHA and all-payer discharge data for 2012–2017 in seven US states. To minimize selection bias, we analyzed records for Veterans admitted to both VHA and non-VHA hospitals in the same year. Using generalized linear models, we adjusted for patient and hospital characteristics.

**Results:**

Following adjustment, VHA admissions consistently had the lowest predicted mean comorbidity scores (4.44 (95% CI 4.34–4.55)) and lowest probability of using the most severe DRG (22.1% (95% CI 21.4%-22.8%)). In contrast, Medicare-covered admissions had the highest predicted mean comorbidity score (5.71 (95% CI 5.56–5.85)) and highest probability of using the top DRG (35.3% (95% CI 34.2%-36.4%)).

**Conclusions:**

More effective strategies may be needed to improve VHA documentation, and current risk-adjusted comparisons should account for differences in coding intensity.

## Background

Documentation of diagnoses is essential for communication between healthcare providers, but it also serves as the basis for risk adjustment in quality measurement and database research. Documented comorbidities can even determine reimbursement in some settings. Past studies have found that Veterans’ comorbidities are more often documented in fee-for-service (FFS) Medicare records compared to records in Veterans Health Administration (VHA) [1–4].

Although some evidence suggested Veterans were sicker when they received care in non-VHA facilities paid by Medicare [3], differing incentives across systems have been proposed as potential reasons for observed coding discrepancies. Non-VHA hospitals bill public payers (i.e., VHA, Medicare and Medicaid) and private insurance companies for distinct inpatient claims. Diagnoses and procedures included in these claims determine the size of payments so providers in non-VHA hospitals have direct incentives to code comprehensively. While VHA may bill Veterans’ private health insurance for care provided for nonservice-connected conditions [5], VHA facilities are largely funded through federal appropriations on a capitated basis per patient.

Coding practices within VHA, however, may have changed over time. The introduction of the Medical Center Allocation System [6], increased attention to risk-adjusted performance reports [7], and expansion of Clinical Documentation Improvement (CDI) programs may have led to more comprehensive coding [8]. Outside of VHA, efforts to improve documentation and coding practices had been taking place for decades [9, 10], and in 2010, the Affordable Care Act created multiple programs linking risk-adjusted quality measures to payment [11]. In addition, enrollment in private managed care plans grew significantly [12, 13], and risk-adjusted payments from public payers to these plans also incentivize comprehensive documentation [14].

Historically, comparisons of care in VHA and non-VHA facilities have been conducted to ensure that Veterans receive high quality care. Efforts are now underway to compare utilization and quality of care in non-VHA facilities that is paid for and provided on behalf of VHA. The Veterans Access, Choice, and Accountability Act of 2014 (Choice) and more recent Maintaining Internal Systems and Strengthening Integrated Outside Networks Act of 2018 (MISSION) allow some Veterans to receive covered services in non-VHA facilities in the community if VHA care does not meet access or quality standards [15]. Community care accounted for over $28 billion (24%) of the budget for medical care in 2023, so researchers and VHA policymakers seek to evaluate the quality of this purchased care. For fair comparisons between VHA-delivered and VHA-purchased care, systematic differences in documentation of comorbidities need to be recognized and accounted for.

Using a unique dataset linking VHA records to all-payer discharge data from state health agencies, we sought to determine whether comorbidity scores and severity levels associated with Veterans’ admissions varied significantly across settings and payers (including non-VHA hospitals paid by VHA, Medicare, Medicaid, commercial insurance, and other sources) from 2012 to 2017 that covered the period of early VHA-purchased care expansion.

## Methods

### Study design

This retrospective study of repeated cross-sectional data examined comorbidity scores and severity levels associated with Veteran enrollees’ admissions in VHA and non-VHA hospitals in seven states.

### Study sample

Veteran enrollees’ all-payer discharge data were obtained from state health agencies and linked to inpatient records from the VA Corporate Data Warehouse Inpatient Encounter files using personal identifiers. The resulting sample included VHA and non-VHA hospitalizations in seven states (AZ, FL, IL, MA, MO, NY, SC). Medicare Severity Diagnosis Related Group (DRG) information was missing from MO all-payer discharge data in years 2016–2017 so these admissions were excluded. Data for all other states included years 2012–2017. Patient-level sociodemographic characteristics, including age, sex, race/ethnicity, marital status, priority group (reflecting military service, disability, and income), and state of residence were obtained from the Assistant Deputy Under Secretary for Health Enrollment Files and the VA Observational Medical Outcomes Partnership Files. Hospital characteristics including number of staffed beds, academic affiliation, and for-profit status were obtained from the Veterans Integrated Service Network Support Services Center and Centers for Medicare and Medicaid Services (CMS) hospital cost reports.

To minimize selection bias of sicker patients being admitted in either setting, we included only Veterans admitted to both VHA and non-VHA hospitals in the same year for the same major diagnostic category (MDC). We also excluded transfers and readmissions (i.e., admissions that occurred within 30 days of discharge from a prior hospitalization).

### Dependent variables

As products of clinical status and coding intensity, the Elixhauser-van Walraven (E-VW) comorbidity score and use of the highest severity level within a DRG family (top DRG) for each admission were the primary outcomes in this study [16, 17]. For each admission, all recorded primary and secondary diagnoses were used to calculate the E-VW score. The E-VW score ranges from -19 to 89, and individual diseases have weights ranging from -7 to + 12. For DRG families with multiple severity levels (e.g., complication or comorbidity, major complication or comorbidity), we specified whether each admission used the highest possible severity level. Admissions with DRGs with only one possible DRG severity level were excluded from the DRG analysis.

### Independent variables

The primary predictor of interest was the setting/payer of each Veteran’s admission: 1. VHA hospital, 2. non-VHA hospital covered by Medicare, 3. non-VHA hospital covered by Medicaid, 4. non-VHA hospital covered by commercial insurance, 5. non-VHA hospital covered by VHA, or 6. non-VHA hospital covered by other payers. Calendar year was also a predictor of interest as we wanted to know whether coding practices changed over time. Age, sex, marital status, Veteran priority group, and state were included as patient-specific sociodemographic characteristics. Admission dates were used to generate a categorical admission sequence variable. For each DRG, the major diagnostic category and an indicator of surgery were also included. Finally, number of staffed beds, academic affiliation, and for-profit status were included as hospital-specific characteristics.

### Statistical analysis

The unit of analysis was the hospital admission. Using generalized linear models, we included setting/payer, categorical calendar year, and the interaction between these indicators as primary predictors. A Gaussian distribution was used for the E-VW model and a binomial distribution was used for the DRG model. In these models, we adjusted for age, sex, marital status, Veteran priority group (reflecting military service, disability, and income), state, admission sequence, MDC, an indicator of surgery, categorical hospital size [18], academic affiliation, and for-profit status. Linear, quadratic, and cubic terms were included for age and admission sequence to allow for non-linearity. Because each Veteran had multiple hospitalizations, standard errors were adjusted for clustering within patient.

For missing covariates, we carried forward patients’ last observed values if recorded previously. We then carried backward observed values if characteristics were available in later encounters. Remaining observations with missing covariates were removed (1.8% of admissions and 1.3% of Veterans). Statistical analyses were conducted using Stata, version 17 (StataCorp).

For interpretation of the results, we computed the predicted mean E-VW scores and predicted probabilities of using the top DRG for each setting/payer over time. To visually compare temporal trends between VHA and non-VHA hospitals, we plotted the predicted mean E-VW scores and probability of using the top DRG with non-VHA hospitalizations grouped.

### Sensitivity analysis

We randomly selected a pair of VHA and non-VHA admissions for each Veteran, as Veterans could have multiple admissions in either setting, and calculated within-patient differences in E-VW comorbidity scores. We then used a generalized linear model to adjust within-patient differences in E-VW scores for admission sequence, year, and MDC. Patient-level demographic characteristics were not included in the model as they were not significant predictors of within-patient differences. Because Veterans could have multiple patient-years in the sample, standard errors were adjusted for clustering within patient.

## Results

The sample included 23,594 Veterans (95% male; mean age 64.7) with 60,942 admissions. Approximately half (51%) of admissions were in VHA hospitals followed by non-VHA-hospitals paid by Medicare (21%), VHA (14%), commercial insurance (6%), Medicaid (3%), and other sources (6%). Admissions in non-VHA hospitals paid for by VHA included medical emergencies and cases in which VHA could not provide care based on availability of services or certain access criteria including lengthy distances to a VHA hospital or long waiting times for care. The six most common major diagnostic categories accounted for 80% of all admissions: Circulatory System (31%), Respiratory System (14%), Mental Diseases and Disorders (13%), Alcohol/Drug Use or Induced Mental Disorders (8%), Nervous System (8%), and Digestive System (6%). Most admissions were non-surgical (86%).

Setting/payer and year were significant predictors (*p* ≤ 0.001) for both E-VW score and use of the top DRG. Medicare was associated with the highest predicted mean E-VW score at 5.71 (95% CI 5.56–5.85) and highest probability of using the top DRG (35.3% (95% CI 34.2%-36.4%)); in contrast the VHA mean comorbidity score was 4.44 (95% CI 4.34–4.55) and probability of using the top DRG was 22.1% (95% CI 21.4%-22.8%). VHA admissions were consistently associated with the lowest mean comorbidity scores and lowest probability of using the most severe DRG levels (Table 1).

**Table 1:** Predicted mean Elixhauser-van Walraven scores and probability of using the most severe DRG level by setting/payer

	2012	2013	2014	2015	2016	2017
VHA, *N* = 31,251^a^						
Mean E-VW Score	4.3 (4.1–4.5)	4.3 (4.1–4.5)	4.3 (4.1–4.5)	4.4 (4.3–4.6)	4.6 (4.4–4.9)	4.8 (4.5–5.0)
Top DRG, % Admissions	18.3 (17.0–19.6)	19.8 (18.6–21.1)	22.2 (20.8–23.5)	22.5 (21.1–23.9)	23.4 (22.0–24.9)	27.0 (25.4–28.5)
Medicare, *N* = 12,782^a^						
Mean E-VW Score	5.4 (5.1–5.7)	5.6 (5.3–5.9)	5.7 (5.4–6.0)	5.9 (5.6–6.2)	5.7 (5.4–6.0)	5.9 (5.6–6.2)
Top DRG, % Admissions	30.5 (28.2–32.8)	33.1 (31.0–35.2)	33.2 (31.0–35.4)	36.0 (33.7–38.3)	38.5 (36.2–40.8)	41.1 (38.7–43.5)
VHA-Paid, *N* = 8,352^a^						
Mean E-VW Score	5.4 (5.0–5.8)	5.4 (5.0–5.7)	5.4 (5.0–5.7)	5.7 (5.3–6.0)	5.5 (5.2–5.9)	5.6 (5.2–5.9)
Top DRG, % Admissions	30.9 (28.0–33.8)	33.0 (30.2–35.8)	33.7 (30.9–36.6)	34.9 (32.0–37.9)	34.9 (32.0–37.7)	38.5 (35.5–41.6)
Commercial, *N* = 3,505^a^						
Mean E-VW Score	5.2 (4.5–5.8)	5.3 (4.8–5.8)	5.4 (4.9–5.9)	5.7 (5.2–6.3)	5.7 (5.2–6.3)	5.3 (4.7–5.8)
Top DRG, % Admissions	26.7 (21.8–31.6)	32.3 (28.4–36.2)	31.4 (27.4–35.3)	36.8 (32.4–41.2)	34.6 (30.3–38.8)	34.5 (29.7–39.2)
Other, *N* = 3,502^a^						
Mean E-VW Score	5.1 (4.6–5.7)	4.6 (4.1–5.1)	5.5 (4.9–6.0)	5.4 (4.9–6.0)	6.0 (5.4–6.6)	5.1 (4.6–5.6)
Top DRG, % Admissions	25.9 (22.0–29.8)	26.1 (22.2–30.1)	28.5 (24.4–32.6)	30.4 (25.8–35.0)	33.1 (28.6–37.6)	36.0 (31.6–40.4)
Medicaid *N* = 1,550^a^						
Mean E-VW Score	5.2 (4.3–6.1)	5.2 (4.4–6.0)	5.6 (4.8–6.3)	6.0 (5.1–6.9)	6.5 (5.5–7.4)	5.9 (4.9–6.9)
Top DRG, % Admissions	25.5 (19.1–31.9)	32.2 (25.5–38.9)	24.7 (18.7–30.7)	32.0 (25.5–38.6)	32.5 (24.2–40.8)	36.8 (27.0–46.6)

Cell values reflect the marginal estimate and 95% confidence interval

*E-VW* Elixhauser-van Walraven, *Top DRG* Highest Severity Level in a Medicare Severity Diagnosis Related Group

^a^A total of 12,882 (21.1%) admissions were not included in the Top DRG analysis because they belonged to a DRG with a single level

**Fig. 1:**
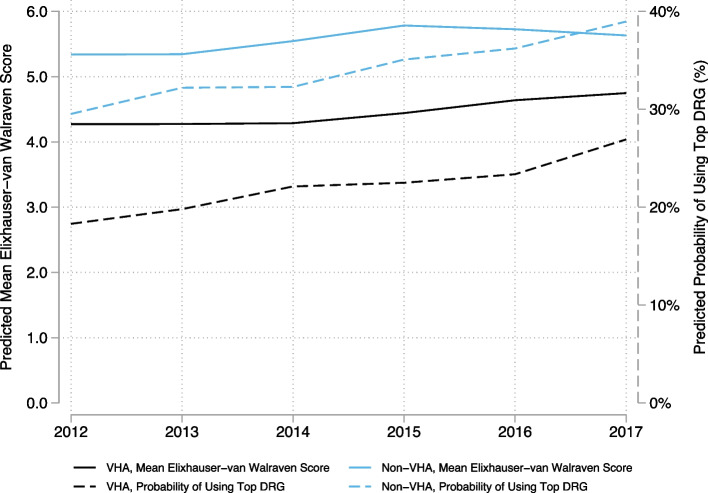
VHA versus non-VHA comorbidity scores and use of top DRG over time. Solid lines reflect Elixhauser-van Walraven scores, and dashed lines reflect the probability of using top DRG (%). VHA, Veterans Health Administration; DRG, Medicare Severity Diagnosis Related Group

Temporal trends across settings/payers, however, were similar with non-significant interactions between setting/payer and year for both metrics (*p* > 0.1). Across all systems/payers, the mean comorbidity score increased from 4.79 (95% CI 4.63–4.95) in 2012 to 5.18 (95% CI 5.01–5.35) in 2017. The overall probability of using the top DRG increased from 23.8% (95% CI 22.8%-24.9%) in 2012 to 32.8% (95% CI 31.6%-34.0%) in 2017. Overall VHA versus non-VHA trends showed persistent relative undercoding (Fig. 1).

**Fig. 2:**
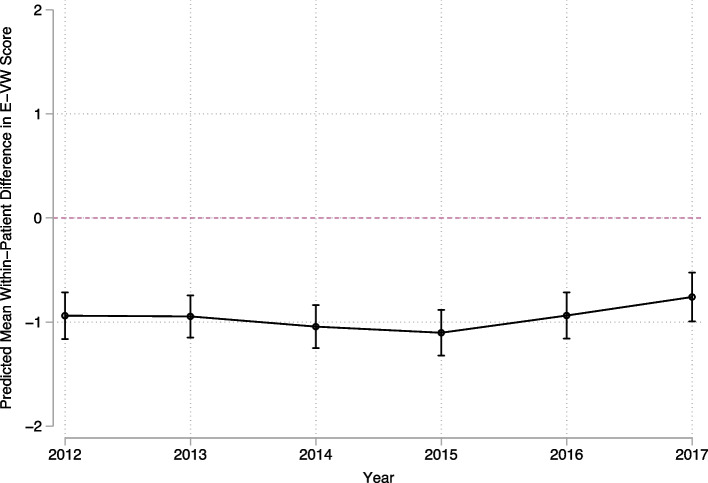
Predicted within-patient differences in Elixhauser-van Walraven scores

In the sensitivity analysis limited to randomly selected pairs of VHA and non-VHA admissions for each patient, the marginal predicted mean within-patient difference in E-VW scores was -0.96 (95% confidence interval -1.05 to -0.87). Calendar year was not a significant predictor of within-patient differences (*p* = 0.39), which demonstrated persistence of lower E-VW scores over time (Fig. 2). Admission sequence was a significant predictor of within-patient differences (*p* < 0.001), which were larger when the VHA admission preceded the non-VHA admission. Patient characteristics associated with admissions included in this sample are included in Table 2.

**Table 2:** Patient characteristics associated with randomly selected paired VHA and non-VHA admissions

	Overall	Medicare	VHA-Paid	Other	Commercial	Medicaid
N	26,032	11,128	7,422	3,087	3,086	1,309
Age, mean (SD)	64.5 (14.0)	70.9 (11.9)	60.6 (13.1)	58.7 (14.0)	60.4 (14.4)	55.0 (11.2)
Sex, N (%)						
Female	1305 (5.0)	340 (3.1)	539 (7.3)	172 (5.6)	172 (5.6)	81 (6.2)
Male	24727 (95.0)	10788 (96.9)	6883 (92.7)	2915 (94.4)	2914 (94.4)	1228 (93.8)
Race/Ethnicity, N (%)						
White, non-Hispanic	17,743 (68.2)	7,600 (68.3)	5,497 (74.1)	1,979 (64.1)	1,991 (64.5)	676 (51.6)
Black, non-Hispanic	5,702 (21.9)	2,432 (21.9)	1,204 (16.2)	799 (25.9)	796 (25.8)	471 (36.0)
Hispanic	1,374 (5.3)	572 (5.1)	365 (4.9)	152 (4.9)	171 (5.5)	114 (8.7)
Other	629 (2.4)	239 (2.2)	229 (3.1)	64 (2.1)	64 (2.1)	33 (2.5)
Unknown	584 (2.2)	285 (2.6)	127 (1.7)	93 (3.0)	64 (2.1)	15 (1.2)
Marital status, N (%)						
Married	9,577 (36.8)	4,404 (39.6)	2,694 (36.3)	1,084 (35.1)	1,174 (38.0)	221 (16.9)
Divorced, Separated, Widowed	12,062 (46.3)	5,318 (47.8)	3,411 (46.0)	1,378 (44.6)	1,294 (41.9)	661 (50.5)
Single, Never Married	4,367 (16.8)	1,396 (12.5)	1,311 (17.7)	619 (20.1)	616 (20.0)	425 (32.5)
Unknown	26 (0.1)	10 (0.1)	6 (0.1)	6 (0.2)	2 (0.1)	2 (0.2)
Priority groups, N (%)						
1,2	9,638 (37.0)	3,734 (33.6)	3,333 (44.9)	1,169 (37.9)	1,126 (36.5)	275 (21.0)
3,4	5,420 (20.8)	2,626 (23.6)	1,257 (16.9)	554 (18.0)	606 (19.6)	374 (28.6)
5,6	8,714 (33.5)	3,662 (32.9)	2,308 (31.1)	1,114 (36.1)	1,020 (33.1)	616 (47.1)
7,8	2,260 (8.7)	1,106 (9.9)	524 (7.1)	250 (8.1)	334 (10.8)	44 (3.4)
Rurality, N (%)						
Urban	19,992 (76.8)	8,593 (77.2)	5,709 (76.9)	2,225 (72.1)	2,314 (75.0)	1,142 (87.2)
Rural	6,040 (23.2)	2,535 (22.8)	1,713 (23.1)	862 (27.9)	772 (25.0)	167 (12.8)

*N* number, *SD* standard deviation

## Discussion

Our goal was to determine whether variation in documentation of comorbidities in VHA and non-VHA admissions had changed over time as the increased emphasis toward performance reporting and growth of CDI programs in VHA may have been influenced coding behaviors. Systematic differences across settings of care are important to understand because documented comorbidities play an important role in risk adjustment for comparisons of quality and performance. Existing studies of VHA care consistently find that quality and safety are as good or better than in other settings [19], but performance advantages may be underestimated if relative undercoding causes VHA-reliant Veterans to appear healthier than peers who use non-VHA care.

Previous studies of documentation of comorbidities have been limited to VHA versus traditional FFS Medicare. Our data is unique as it captured utilization under both FFS Medicare and Medicare Advantage (MA) plans as well as utilization covered by VHA, Medicaid, commercial insurance, and other payers. Previous comparisons also focused on distinct diagnoses and risk scores, and this data includes DRGs which have a direct relationship to hospital reimbursement. A study utilizing the Healthcare Cost and Utilization Project National Inpatient Sample found that use of the highest severity level increased over time for 15 of the top 20 most reimbursed DRG families despite reductions in risk-adjusted mortality [20]. This observed change in coding was associated with $1.2 billion in increased payments. While we found a small increase in E-VW scores and notable changes in use of top DRG severity levels in VHA hospitals over time, differences between VHA and non-VHA hospitals were persistent.

VHA launched a national CDI program in 2013, and half of VHA medical centers had implemented a CDI program by 2016 [21]. VA internal audits in 2016, however, found that correct evaluation and management (E/M) codes were only used in 60% of encounters nationwide [21]. VA officials attributed errors to lack of provider training, lack of emphasis on the importance of accurate coding, and lack of time for careful coding. The establishment and growth of VHA CDI programs likely contributed to the observed increase in E-VW scores and use of the top DRG over time; however, the persistent differences in these metrics between VHA and non-VHA hospitals suggest that different strategies may be necessary to improve uptake of best practices. Physicians are ultimately responsible for entering diagnosis codes and providing supporting documentation, but they may see these activities as distractions from clinical care. Consequently, the importance of buy-in and rapport between physicians and CDI specialists has been highlighted as a crucial factor for success [10]. One study examined the benefit of in-person, verbal communication between CDI specialists and clinicians and found significant improvements in time to resolution [22].

It is also possible that efforts to improve coding behaviors within VHA will not be sufficient to offset the effects of financial incentives on coding intensity outside VHA. To account for well-established differences in coding intensity observed between MA organizations and FFS providers, the CMS applies a coding pattern adjustment, reducing risk-based payments to MA plans by 5.9% [23]. This adjustment is the minimum amount required by the American Taxpayer’s Relief Act of 2012, however, and larger adjustments may be warranted [24]. Methods to estimate an appropriate coding pattern adjustment for MA plans have been proposed, but there is no consensus regarding the best method [25]. Future studies comparing VHA and non-VHA risk-adjusted quality and performance may consider developing risk-score adjustment methods that reduce bias from differences in coding intensity between VHA care and non-VHA care. Compared to other Veterans, those who use VHA have a higher prevalence of chronic physical and mental health conditions [26]. VHA-reliant reliant also have lower self-reported health [27]. Consequently, such an adjustment would likely be conservative.

### Limitations

Our study was limited to seven states with data spanning 2012 to 2017, so these findings may not be representative of current admissions on a national level. Although we adjusted for admission sequence to account for clinical progression, Veterans may receive non-VHA care when they are sicker. Of the Veterans admitted twice in the same year, 15–17% were admitted to both VHA and non-VHA hospitals. The study sample may not be generalizable to the general populations of Veterans with hospitalizations; however, restricting the sample to Veterans admitted in both settings under the same major diagnostic category allowed us to remove some selection bias. Finally, we did not have access to outpatient diagnoses. Billing for outpatient E/M services, however, creates similar incentives for non-VHA clinicians to code diagnoses intensively as the number and complexity of problems affects the level of service and reimbursement.

## Conclusion

These data suggest that relative undercoding has persisted in VHA and highlight differences between VHA hospitals that are funded as part of an integrated delivery system and non-VHA hospitals that may document comorbidity for payment. While risk scores and DRG severity increased in VHA over time, the difference between VHA and non-VHA hospitals was persistent, suggesting that internal efforts to improve documentation may not be sufficient. Future studies may need to apply alternative strategies, including coding pattern adjustments, to ensure fair comparisons of quality and performance.

## Data Availability

Data from this report will not be available to others because data access is restricted under a memorandum of understanding between the study team and each state agency that provided all-payer discharge data. Statistical code will be made available to others by email request to the corresponding author.

## Abbreviations

CDI: Clinical Documentation Improvement
CMS: Centers for Medicare and Medicaid Services
DRG: Medicare Severity Diagnosis Related Group
E/M: Evaluation and management
E-VW: Elixhauser-van Walraven
FFS: Fee-for-service
MA: Medicare Advantage
MDC: Major diagnostic category
MISSION: Maintaining Internal Systems and Strengthening Integrated Outside Networks Act of 2018
VHA: Veterans Health Administration
